# A Multidimensional View on Social and Non-Social Rewards

**DOI:** 10.3389/fpsyt.2020.00818

**Published:** 2020-08-19

**Authors:** Magdalena Matyjek, Stefanie Meliss, Isabel Dziobek, Kou Murayama

**Affiliations:** ^1^ Berlin School of Mind and Brain, Humboldt-Universität zu Berlin, Berlin, Germany; ^2^ Department of Psychology, Humboldt-Universität zu Berlin, Berlin, Germany; ^3^ School of Psychology and Clinical Language Sciences, University of Reading, Reading, United Kingdom; ^4^ Research Institute, Kochi University of Technology, Kochi, Japan

**Keywords:** social reward, non-social reward, reward dimension, primacy, tangibility, familiarity, reinforcement learning

## Abstract

Social rewards are a broad and heterogeneous set of stimuli including for instance smiling faces, gestures, or praise. They have been widely investigated in cognitive and social neuroscience as well as psychology. Research often contrasts the neural processing of social rewards with non-social ones, with the aim to demonstrate the privileged and unique nature of social rewards or to examine shared neural processing underlying them. However, such comparisons mostly neglect other important dimensions of rewards that are conflated in those types of rewards: primacy, temporal proximity, duration, familiarity, source, tangibility, naturalness, and magnitude. We identify how commonly used rewards in both social and non-social domains may differ in respect to these dimensions and how their interaction calls for careful consideration of alternative interpretations of observed effects. Additionally, we propose potential solutions on how to adapt the multidimensional view to experimental research. Altogether, these methodological considerations aim to inform and improve future experimental designs in research utilizing rewarding stimuli, especially in the social domain.

## Social and Non-Social Rewards

Rewards are desired, appetitive, and positive outcomes of motivated behavior that *can* increase and maintain the frequency and strength of the behavior they are contingent on (1). They often serve as reinforcers, i.e. positive (or in other cases negative) stimuli or events that *actually* change the probability of that behavior’s occurrence or its strength in the future (2). Because humans do not live in isolation, many rewarding experiences stem from social interaction and relationships. *Social* *rewards* are a broad set of stimuli, which instigate positive experiences involving other people, including a vast repertoire of verbal and non-verbal behaviors, gestures, and feelings (3) such as a smile (4), praise (5), a thumbs-up (6), acquisition of good reputation (7), etc. However, despite the considerable heterogeneity of social rewards and abundance of research utilizing them, it is not clear what constitutes rewards as social and there has been surprisingly little systematic discussion on how we can conceptualize them. Nevertheless, regardless of lacking a clear definition of social rewards, there is a large body of literature discussing them in relation to non-social ones.

Social rewards have been studied by two different lines of research. The first line of research aims to address the “privileged” nature of social rewards, arguing that there are dedicated, special mechanisms that subserve social functioning, including social rewards. These studies often contrast them against non-social rewards to demonstrate if and how they are processed differently from non-social environmental rewards. For example, autism, which is characterized by pervasive social impairments (8), has been taken as an example of atypical responsiveness to social cues. Researchers have hypothesized impaired processing of social, and preserved processing of non-social rewards [social motivation hypothesis; Chevallier et al. (9)] and have been testing this prediction by comparing responses to social and non-social rewards [for a review, see Bottini (10)]. The comparison is also common in other fields with non-clinical populations [e.g., Kohls et al. (11)].

Another line of research has indicated that social and non-social rewards may be processed in a similar manner. This is supported by economic theories proposing that behaviors stem from the desire to maximize the ratio of rewards to costs (12) and this applies to non-social as well as to social rewards [social exchange theory, Thibaut and Kelley (13)]. Indeed, many studies investigating the neural basis of reward processing found that social and non-social rewards are processed in the same brain areas of what is referred to as the *reward network* [i.e. a cortico-basal ganglia circuit, Haber and Knutson (14)], especially in the striatum, supporting the assumption of an “extended common currency schema” (15). However, researchers have also emphasized specific activity differences in line with the idea of “social-valuation-specific schema” (15), which assumes dedicated brain circuits for social rewards. For instance, a study comparing the rewarding properties of receiving money or positive social feedback found that both rewards activated the striatum, especially the left nucleus caudate, and that this region also showed a linear activity increase towards both reward values (7). A reanalysis of the same data using machine learning, however, yielded a fairly small correlation between classifier weights for social and monetary rewards, suggesting that only a subset of neurons in the caudate nucleus encodes both rewards, whereas also distinct populations of neurons are involved for social and for non-social rewards separately (16). Thus, although both types of rewards can be processed in similar structures of the reward network in the brain [e.g. Izuma et al. (7); Spreckelmeyer et al. (4); Wake and Izuma (16)]; Smith et al. (17); Levy and Glimcher (18); Lin et al. (19), there has also been accumulating evidence for differences in neural processing between social and non-social rewards [e.g. Izuma et al. (7); Smith et al. (17); Sescousse et al. (20); for a recent review of literature discussing overlaps and differences in neural processing of social and non-social rewards, see Ruff and Fehr (15)].

These studies suggest that there are both similarities and differences in neural processing between social and non-social rewards. However, we argue that research comparing social and non-social rewards often neglects important dimensions that can be conflated with the sociality dimension. For example, comparing brain responses to receiving a smile or money may potentially reveal a difference between social and non-social rewards as well as between intangible and tangible rewards. In this article, we propose a more comprehensive, multidimensional view on rewards in experimental settings, which allows more informed and better-controlled comparisons of social and non-social rewards.

## Dimensions of Rewarding Stimuli

**Figure 1:**
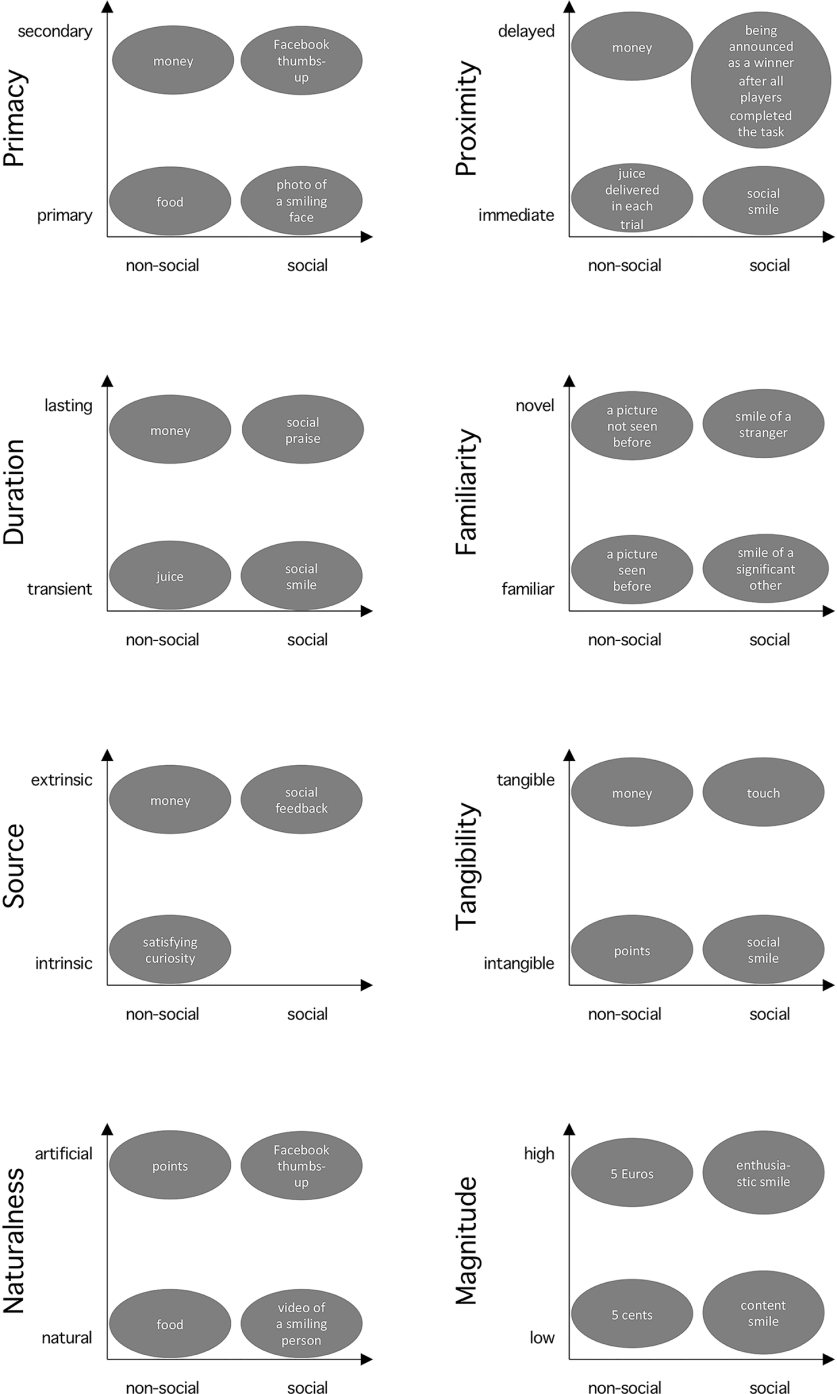
Interplay of the sociality and other reward dimensions. The x-axis represents the sociality dimension. The provided cases illustrate examples of rewards used in psychology and neuroscience placed along the dimensions discussed in this article. The spatial distance between the cases does not directly depict differences in their rewarding value.

Research contrasting social and non-social rewards implicitly assumes a binary categorization of those rewards. However, monetary reward is considered as non-social, but money could be regarded as a “social construct” in the sense that it would not exist without society and a collective agreement of their function [social constructionism, e.g. Galbin (21)]. Thus, binary categorization of social and non-social may be an oversimplification, and a continuous dimension may provide a more accurate conceptualization. Moreover, we suggest that there are other dimensions to describe rewards, e.g. tangibility and primacy, and that considering them can offer alternative interpretations of observed differences between social vs. non-social rewards. This section describes these dimensions of rewarding stimuli (see **Figure 1** for an overview). Our goal is not to provide a complete list of all possible dimensions, but to outline the scope of this multidimensional view with several examples, which we consider particularly relevant for social vs. non-social reward processing: primacy, temporal proximity, duration, familiarity, source, tangibility, naturalness, and magnitude. Importantly, we discuss how each of these dimensions interacts and confounds with social vs. non-social dimension.

### Primacy

Primacy is a dimension categorizing rewards [after theories of operant conditioning, Skinner (22)] depending on whether they stem from innate or biologically pre-programmed reinforcing states (hunger satisfied by food or mother’s closeness satisfying the need for touch of an infant) on one hand (i.e. primary rewards), or having rewarding properties through learned or acquired associations with primary reinforcers (money as a means to acquire food, a Facebook thumbs-up to gain social appreciation) on the other hand [i.e. secondary rewards; Delgado et al. (23)]. Thus, primary and secondary rewards can be found in both, social (touch, thumbs-up) and non-social (food, money) domain. Studies have shown that even though there is a partial overlap in the ventromedial prefrontal cortex (vmPFC) representing the anticipatory value of primary and secondary rewards (18, 24), there is also additional activity specific to primary (i.e. hypothalamic regions) and secondary rewards [i.e. posterior cingulate cortex; Levy and Glimcher (18)], respectively. Since primacy can be linked to distinct neural processing, it is important to choose rewards of the same primacy nature when comparing social and non-social ones.

### Temporal Proximity

Temporal proximity describes the temporal relationship between motivated behavior and reward reception (e.g., immediate vs. delayed). There is evidence that they are processed distinctly in the human brain [e.g., Ballard and Knutson (25); for a review, see Bermudez and Schultz (26)]. Specifically, midbrain, striatum, frontal cortex, and amygdala are all sensitive to time of reward occurrence (soon or later). Moreover, temporal discounting may lead to a preference for sooner smaller compared to later larger rewards. Social rewards are usually delivered immediately at the end of the trial in the form of a smile or social feedback, aligning simultaneous reception and consumption of reward. However, in the non-social domain, there is often a difference between reward reception in an experimental trial (e.g. a picture of a coin) and the actual consumption of the reward after the experiment (i.e., receiving the physical money). Note that sometimes the amount of points won in trials is not even directly translated to actual money gains (27). Thus, comparing social rewards with non-social rewards may trigger brain responses reflecting differences in the temporal proximity dimension in addition to the sociality dimension.

### Duration

The dimension of duration distinguishes between lasting and transient rewards. Unlike transient rewards (consumed/appreciated while presented), lasting ones may entail accumulation over time, which affects economic decision making and activity in vmPFC (28). While social ones most often are transient (a smile lasts only while presented, but praise may have longer-lasting effects generating feelings of appreciation), non-social rewards are more dependent on the experimental context. For example, money received in a task is still available after the end of the experiment, whereas juice delivered on a trial-by-trial basis is immediately consumed. Thus, when comparing social and non-social rewards, duration needs to be considered to avoid confoundedness.

### Familiarity

Familiarity differentiates novel from familiar stimuli and is signaled in the striatum and the midbrain (29). While novelty is rewarding in non-social stimuli (29), it may be the opposite in the social domain, where familiar and socially relevant faces are more rewarding than faces of strangers (30). In fact, it has been shown that familiar faces are processed differently than faces of unknown people, due to different visual representations stored in memory, personal knowledge, and personal relevance (31). Furthermore, “familiarity” in the context of social rewards has multi-faceted meanings and there may be qualitative differences between familiarity with relatives, celebrities, and experimentally learned individuals (31), which can potentially lead to inconsistencies through differential engagement in experimental tasks (32). Altogether, familiarity may modulate social and non-social rewards differently, which should be considered in study designs.

### Source

Source relates to whether the rewarding nature originates internally (i.e. intrinsically within a person, e.g. feeling curious) or externally (i.e. extrinsically by receiving food or praise). While psychological theories consider them as distinct [e.g., Deci and Ryan (33)], neuroscientific studies show that rewards from both sources activate the reward network (34), with additional brain regions specific for intrinsic rewards [the anterior insula; Lee (35)]. This can be a potential confound for the sociality dimension, as non-social rewards could stem from both sources (satisfying curiosity or receiving money), but social rewards are by definition extrinsic as provided by others (e.g. social feedback).

### Tangibility

Tangibility refers to the property of a stimulus to be touched or consumed, with more abstract stimuli being less tangible. Studies suggest differential reinforcing and motivating effects of tangible and intangible stimuli (36), often *via* differential engagement of intrinsic and extrinsic motivation (37). For example, in a study with tangible monetary and intangible verbal rewards on intrinsic motivation, only the latter showed positive and prolonged effects (38). Because social rewards are most often intangible (like verbal praise) and non-social rewards are tangible (e.g. money), the interaction of sociality and tangibility is a potential confound.

### Naturalness

Some studies use natural stimuli such as chocolate (18) or verbal praise (39) as rewards, whereas other studies use more arbitrary, symbolic stimuli such as Facebook thumbs-up icon (6) or a picture of a coin (11). Naturalness is especially important for social rewards. For example, there is an increasing number of studies using avatars [e.g. Kim et al. (40)] and cartoon representations of faces [e.g. Gonzalez-Gadea et al. (41)], which convey the social nature through the resemblance to their natural equivalences (faces). In fact, computer-generated and natural faces have been shown to elicit similar emotional processing in the amygdala, but also differential activation in the fusiform face area (42). Again, the interaction of sociality and this dimension should be considered and controlled for by choosing both social and non-social rewards to be either natural or representational.

### Magnitude

The magnitude of a reward can be defined as the extent of its objective and subjective value. Studies have shown that activity in the ventral striatum correlates with the objective magnitude of both monetary [increasing amounts; Knutson et al. (43)] and social rewards [happy face expressions with increasing intensity level; Spreckelmeyer et al. (4)], and vmPFC correlates with the subjective magnitude of rewards (19). Critically, rewards with higher magnitude are likely to elicit larger responses in wider areas of the brain in comparison to rewards with lower magnitude [e.g. Smith et al. (44); Diekhof et al. (45)]. Differences in magnitude between rewards should thus be avoided to allow interpretation of the observed effects in terms of social vs. non-social (and not low vs. high magnitude).

In addition to the dimensions above, some other aspects contrast social rewards against other rewards. For example, social stimuli are usually complex and can be more ambiguous than non-social ones: The same smile may be interpreted as a friendly reaction or as a ridicule, depending on the context. Thus, it is important to take into account biases in the interpretation of ambiguous social stimuli linked to internal states [e.g. negativity bias in depressive states; Dai et al. (46)]. Also, psychological traits and conditions [like autistic traits and social anxiety; Cox et al. (47) and Cremers et al. (48), respectively] have been shown to modulate responses to social rewards specifically. Likewise, visual complexity may introduce altered processing: Non-social rewards are often less visually complex than their social counterparts (6, 49), introducing a perceptual bias and neural differences (50). Furthermore, it may be more challenging to uniformly induce a rewarding value of social stimuli than of non-social ones, as the rewarding value of social stimuli depends on a certain context around participant and reward. In fact, a smiling face seen on the screen can be rewarding for a participant performing a task only when they believe to some extent that this smile is contingent on their action, as it happens in natural interactions. Simply instructing participants that a smiling face indicates positive feedback might not make it sufficiently socially rewarding; this requires a perceived social context between the participant and the person on the screen, entailing that “social interaction must not inherently be rewarding due to the appearance of positive social stimuli” [Krach et al. (51), p.1]. Although some studies suggest that bottom-up processes are involved in the privileged processing of social stimuli (52), for a stimulus to be *socially rewarding*, it is not enough to be a representation of human likeness/gesture carrying positive feedback. Social rewards require the component of intention and direction from the observer to the observed, even if there is no direct (face-to-face) interaction between those two. In fact, one could consider social rewards that are delivered without a social visual stimulus. For example, in Kujawa et al. (53) participants saw a green checkmark (abstract symbol) as signifying social acceptance, a salient social reward (54). This is especially important considering recent attempts to bring experimental research closer to reality, which includes the use of dynamic stimuli (55, 56) and implementing a second-person approach in (neuroscientific) research on social cognition (57). Although instantiating social context may come at the cost of losing experimental control, some promising designs aiming to ensure ecological validity and experimental control have been proposed [e.g. Drimalla et al. (58)].

## Implications of the Multidimensional View on Rewarding Stimuli in Experimental Designs

As discussed, rewards can be described on multiple dimensions and each of them can be linked to different neural correlates and psychological processes. Thus, research interested in comparing social against non-social rewards should carefully control for other dimensions that may conflate the dimension of interest instead of ascribing the observed effects to a single one, like sociality. However, research has rarely considered these additional aspects of rewards [but see the discussion of primacy and tangibility of money and juice, Kim et al. (24); or praise, Wake and Izuma (16)]. For example, many studies simply compare smiling faces and monetary outcomes to examine the differences of social vs. non-social processing (59–62). However, both outcomes differ not only on the social – non-social dimension, but also in terms of their 1) tangibility: a smile is not tangible, but money as a reward in the form of coins and notes is; 2) primacy: a smile is a primary reward^1^, money is secondary; 3) proximity and duration: a smile is immediate and transient (its rewarding value lasts as long as its exposure), whereas money is lasting and distant, as it will be delivered at the end of the experiment. Hence, from this multidimensional perspective observed differences between responses to smiles and money cannot be fully ascribed to the social vs. non-social contrast but could also stem from differences in tangibility, primacy, proximity, and duration.

How can empirical research overcome these potential limitations? One strategy is to incorporate these dimensions as additional factors in an experimental design [e.g. visual complexity in Pfabigan et al. (50)]. However, this exponentially increases the number of conditions, which substantially boosts the length of the experiment and/or required sample size. An alternative solution is to use stimuli that match in other dimensions than sociality as much as possible. Previous research has shown that pleasant odors can engage the reward circuits (64, 65, 66) which could be used in a comparison with social rewards like smiling faces. Both rewards would be balanced in terms of temporal proximity (both immediate), tangibility (both intangible), source (both external), and they can be matched with respect to their primacy, duration, familiarity, naturalness, and magnitude. Another approach could be to condition social and non-social rewards with neutral stimuli. For instance, Lehner et al. (67) matched reward magnitude of chocolate, money, and social smile with thumps-up using a willingness-to-pay paradigm and later paired them with neutral stimuli (matched in color, luminance, and complexity) to then measure the response to those stimuli. Finally, another potential solution would be to assess other dimensions as much as possible (e.g. using subjective ratings) and statistically control for these effects in the analysis. This strategy can also address potential individual differences in the interpretation of social stimuli.

Another implication of this multidimensional view is noteworthy for one of the most widely-used paradigms that compare social and non-social rewards: Monetary [MID; Knutson et al. (43, 68)] and Social [SID; Spreckelmeyer et al. (4)] Incentive Delay tasks. In these tasks, participants are presented with a cue indicating possible outcomes in a given trial: a gain or loss, or no outcome (control condition). After a variable anticipation delay, they perform a task after which feedback (i.e. the amount of reward or punishment) is delivered depending on participants’ performance. An advantage of the incentive delay paradigm is that it allows targeting both reward anticipation triggered by an incentive cue indicating a possible future reward, and reward reception, elicited with a rewarding stimulus after task performance (43, 68). It has been shown that both phases (anticipation and reception) involve different brain regions and they are modulated differently by the domain of rewards (social and non-social), with reception being more domain-specific than anticipation (69). This paradigm has intuitive appeal to contrast social and non-social rewards, but our multidimensional view suggests the potential difficulty in interpreting the results in terms of anticipation and reception, especially in the context of comparing social and non-social rewards.

For example, Kohls et al. (59) used a picture of a smiling face as both incentive cues and rewards in the SID task. However, a smile is an immediate reward (participants are being smiled at the moment), which entails that as an incentive cue it triggers not only anticipation as intended, but also reception of this reward. Moreover, in the MID task, a picture of a coin is normally presented as a signal that the trial was successful and thus participants receive a monetary reward. However, in reality, participants receive physical money at the end of the experiment, not immediately after each trial (money is a distant reward in such settings). Hence, a picture of a coin intended to represent a reception of reward may actually trigger another anticipation. In other words, when considering the dimension of temporal proximity, for both cases, the distinction between the reward processing phases becomes rather arbitrary. Confounding these two factors (reward processing phases and domain) has serious consequences on how we should interpret the results because both phases are associated with distinct brain areas (70). Disentangling of those factors could be achieved by using neutral, non-rewarding incentive cues to trigger anticipation [e.g. Matyjek et al. (71)], or by matching social and non-social rewards on the temporal proximity dimension (i.e. immediate vs. delayed rewards). For instance, to match social rewards, which are often immediate (e.g. a smile), their non-social counterparts can be delivered on a trial-by-trial basis, e.g. in form of juice (24) or direct online bank transfers. Similarly, to match non-social rewards, which have often delayed reception (e.g. money), the social condition could include trial-by-trial symbolic indications of positive feedback, which translate into social appreciation at the end of the experiment in a form of positive adjectives describing the participant (7), given by an “observer”.

At a broader level, one important implication of the proposed multidimensional perspective is that it highlights a more nuanced relationship between social and non-social rewards than what researchers have previously assumed. As indicated earlier, while many studies seek neural correlates specialized to social processes, another body of literature focuses on the similarities among different types of rewards (including social), suggesting that there is a common valuation network in the brain. These two lines of research seem contradictory: One argues that social and non-social rewards are different and the other suggests that they are the same. However, the proposed multidimensional view provides a simple integration (see also Murayama (34), in the context of the distinction between intrinsic and extrinsic rewards). While social and non-social rewards are both reinforcers with the potential to guide behavior, their differential effects are (at least in part) attributable to properties on other dimensions on which rewards can be described (e.g., temporal proximity, familiarity, etc.). Using the multidimensional view as a starting point, we can thoroughly reflect upon mechanisms underlying the processing of social rewards, being able to go beyond the simple assertion that social rewards and non-social rewards are either similar or different.

## Author Contributions

All authors contributed to the article and approved the submitted version.

## Funding

This study was supported by funding from the Berlin School of Mind and Brain, Humboldt-Universität zu Berlin, the German Academic Exchange Service (Deutscher Akademischer Austauschdienst; DAAD), JSPS KAKENHI (16H06406, 18H01102, and 18K18696), the F. J. McGuigan Early Career Investigator Prize, the Jacobs Foundation Advanced Fellowship and the Leverhulme Trust (RL-2016-030).

## Conflict of Interest

The authors declare that the research was conducted in the absence of any commercial or financial relationships that could be construed as a potential conflict of interest.

## Footnotes

1 In this article, we consider smile as a primary reward as suggested by infants’ preference for smiling faces (63), but other interpretations are possible.
